# Donor-derived invasive aspergillosis after kidney transplant

**DOI:** 10.1016/j.mmcr.2018.07.004

**Published:** 2018-07-17

**Authors:** Maricela Valerio, Marina Machado, Santiago Cedeño, Maria Luisa Rodríguez, Fernando Anaya, Antonio Vena, Jesús Guinea, Pilar Escribano, Emilio Bouza, Patricia Muñoz

**Affiliations:** a Clinical Microbiology and Infectious Diseases Department, Hospital General Universitario Gregorio Marañón, Doctor Esquerdo 46, 28007 Madrid, Spain; b Instituto de Investigación Sanitaria del Hospital Gregorio Marañón, Doctor Esquerdo 46, 28007 Madrid, Spain; c Nephrology Department, Hospital General Universitario Gregorio Marañón, Doctor Esquerdo 46, 28007 Madrid, Spain; d Medicine Department, School of Medicine, Universidad Complutense de Madrid, Pza, Ramón y Cajal, s/n. Ciudad Universitaria, 28040 Madrid, Spain; e CIBER Enfermedades Respiratorias-CIBERES (CB06/06/0058), Madrid, Spain

**Keywords:** Invasive fungal infections, Invasive aspergillosis, Solid organ transplant, Donor-derived infections, Kidney transplant

## Abstract

The risk of transmission of infectious diseases from allograft to recipient is well known. Viruses and bacteria are the most frequent causes of transmissible infections. Donor-derived invasive aspergillosis is rare and occurred under particular circumstances. We report 2 cases of kidney transplant recipients who acquired aspergillosis from a single donor.

## 1 Introduction

The risk of transmission of infectious diseases from allograft to recipient is well known, although nowadays uncommon [1]. Viruses and bacteria are the most frequent causes of transmissible infections, with fewer than 25% of donor-derived infections due to fungi [2], mostly *Candida* spp. [3].

Cases of donor-derived invasive aspergillosis (IA) in transplant patients are unusual [4], [5]. Here we report 2 cases of kidney transplant recipients who acquired aspergillosis from a single donor.

## 2 Case

### 2.1 Donor

The grafts came from a 50-year-old man with a history of alcoholic cirrhosis, several episodes of spontaneous bacterial peritonitis requiring antimicrobial treatment, and a recent admission due to acute alcoholic hepatitis that was treated with high-dose corticosteroids. Fifteen days after discharge he was readmitted with acute liver failure that again required corticosteroids and a relapse of *C. difficile* infection. On day 7 after admission, he presented with fever and an acute neurologic event requiring ICU admission and intubation. A CT scan demonstrated bilateral intraparenchymal hematomas with uncal herniation and new bilateral lung infiltrates.

Bronchoalveolar lavage (BAL) culture revealed extended-spectrum beta-lactamase producing (ESBL) *Klebsiella pneumoniae* that was treated with meropenem. The patient died on his fifth day at ICU. Death was attributed to a cerebral hemorrhage resulting from severe liver failure with massive bronchoaspiration.

His potential as a kidney donor was based on a renal ultrasound that showed a simple cyst in the cortex of the left kidney (1.3 cm) and a Doppler ultrasound image that revealed adequate vascular flow in both kidneys. Accordingly, the transplant committee accepted his kidneys for transplant. His liver and heart were not used as grafts.

### 2.2 Recipient 1

He was a 56-year-old man who had received a liver transplant (LTx) 15 years earlier, for which he took cyclosporine A (CyA) and mycophenolate mofetil (MMF). His general progress was good, and his graft function adequate. Three years after the LTx he experienced hepatitis C relapse and was treated with interferon and ribavirin. Since then, the patient has maintained good liver function.

He developed end-stage renal disease (ESRD) due to hepatitis C associated–membranoproliferative glomerulonephritis. Residual diuresis was 100 mL/24 h, and he had been receiving hemodialysis for the past 36 months. He received a kidney transplant from a deceased donor with 3 HLA mismatches. The pretransplant biopsy graft contained 32 glomeruli, with no involvement of the vascular or tubulointerstitial compartment.

The surgical procedure was uneventful, and the immunosuppression regimen included basiliximab, methylprednisolone, CyA, and MMF. Cold ischemia time was 6 h, and, despite good graft perfusion, he had delayed graft function and required hemodialysis on day 2 after transplantation.

Given the donor's history of respiratory infection, empirical ceftriaxone was started from day 0 to day 3 after surgery and was then changed to meropenem as soon as ESBL *K. pneumoniae* was identified in a BAL culture.

### 2.3 Recipient 2

He was a 56-year-old man with ESRD secondary to nephroangiosclerosis. He had been on hemodialysis for 3 years. Residual diuresis was 1500 mL/24 h, and he underwent deceased donor kidney transplantation with 3 HLA mismatches. The pretransplant biopsy graft contained 39 glomeruli, with no involvement of the vascular or tubulointerstitial compartment.

His immunosuppressive treatment included basiliximab, methylprednisolone, tacrolimus, and MMF. The surgical procedure was uneventful, and cold ischemia time was 10 h. He had delayed graft function despite good graft perfusion. He also received empiric ceftriaxone from day 0–3 post-transplant, and was then switched to meropenem.

### 2.4 Transplant procedure and follow-up

At the time of kidney explantation and transplantation, the urologist noticed that one of the kidneys had a cyst. However, kidney biopsy specimens stained using rapid techniques in the pathology and microbiology departments were immediately reported as “normal”, and transplantation was performed.

On day 1 after surgery, a filamentous fungus started to grow in the culture plate of the donors’ respiratory sample (BAL). It was later identified as *Aspergillus fumigatus*. With this information, analysis of the previous renal biopsies reassessed with specific fungal stains (calcofluor white) revealed filamentous fungi, which were subsequently confirmed by the histopathologist.

Despite the fact that both recipients were in a stable clinical situation and given the potential presence of IA, elective nephrectomy was performed on day + 3 after transplant in both cases. Surgery was uneventful, and the graft and vascular sutures were resected. In patient 1, the gross appearance of the kidney and the transverse section was normal. Samples of the cortex, medulla, hilum, and vascular sutures were sent for culture and histology.

In patient 2, the kidney presented several parenchyma cavitations. Samples of the same areas were sent for culture and histology.

In both cases, *A. fumigatus* was isolated in samples from the cortex and vascular sutures. Tissue invasion was confirmed, and real-time PCR was positive for *Aspergillus* spp. in both cases. Chest radiographs were normal in both cases after graft extraction. Serological and urinary detection of [1], [3] β-D-glucan (BDG) was performed (positive cut-off ≥80 pg/mL). BDG levels in blood and urine were as follows: Patient 1, 411 pg/mL in blood and 1647 pg/mL in urine on the first day after transplant; Patient 2, 433 pg/mL in blood and 1218 pg/mL in urine on the first day after transplant (Table 1).

**Table 1:** Characteristics of organ recipients and peritransplant microbiology and medications.

	**Patient 1: 56/Male**	**Patient 2: 56/Male**
**Clinical History**		
** *Underlying disease***	ESRD secondary to hepatitis C–associated membranoproliferative glomerulonephritis	ESRD secondary to nephroangiosclerosis
** *Comorbidities***	Liver transplant in 2000 (15 years previously) due to decompensated cirrhosis.	Primary hypothyroidism; Hiatal hernia; Peptic ulcer disease
** *Transplant***	Renal graft	Renal graft
**Microbiology results**		
** *Donor***	ESBL *Klebsiella pneumoniae* and *Aspergillus fumigatus* (BAL culture)	ESBL *Klebsiella pneumoniae* and *Aspergillus fumigatus* (BAL culture)
** *Recipient***	*Aspergillus fumigatus* (samples from the cortex and vascular sutures)	*Aspergillus fumigatus* (samples from the cortex and vascular sutures)
**Laboratory results**		
** BDG pg/mL**		
*** Blood (day post-transplant)***	411 (+2) – 116 (+8) – 103 (+10) – 50 (+23)	433 (+2) – 141 (+6) – 200 (+18) – 58 (+30)
*** Urine (day post-transplant)***	1647 (+2)	1218 (+2)
** GM ng/mL**	0.59	0.88
**Histopathology**		
	Renal aspergillosis with cortical and medullary intraparenchymal microabscesses	Renal aspergillosis with intraparenchymal microabscesses in the cortical and medullary region
**Medications**		
** *Immunosuppression***	Basiliximab, methylprednisolone, CyA, and MMF	Basiliximab, methylprednisolone, tacrolimus, and MMF
** *Antimicrobials***	Ceftriaxone and meropenem	Ceftriaxone and meropenem
** *Antifungal treatment***	Liposomal amphotericin B (3 mg/kg/day)	Voriconazole (6 mg/kg/12 h)

**ESRD:** End-stage renal disease; **BDG**: 1,3-ß-D-glucan; **GM**: galactomannan; **BAL**: bronchoalveolar lavage; **ESBL**: Extended-spectrum β-lactamases; **CyA:** cyclosporine A**; MMF**: Mycophenolate mofetil. **ESRD:** End-stage renal disease; **BDG**: 1,3-ß-D-glucan; **GM**: galactomannan; **BAL**: bronchoalveolar lavage; **ESBL**: Extended-spectrum β-lactamases; **CyA:** cyclosporine A**; MMF**: Mycophenolate mofetil.

*A. fumigatus* was genotyped directly from positive PCR samples (BAL sample from the donor and renal biopsies from recipients) using a highly discriminatory microsatellite procedure Short Tandem Repeats Aspergillus fumigatus (STRAf). Clinical isolates were not stored and consequently were not genotyped. All genotypes found were identical and showed the same allele composition for the 9 markers.

Antifungal treatment was initiated in both cases before extraction. In patient 1, liposomal-amphotericin B (3 mg/kg/day) was preferred in order to minimize drug interactions with azole derivatives and the immunosuppressive therapy he received for the LTx. Voriconazole (6 mg/kg/12 h) was chosen for patient 2 on day 1, followed by 4 mg/kg/12 h, with weekly therapeutic drug monitoring and subsequent dosage adjustment if needed.

Both patients were followed for 1 year and remain asymptomatic.

We report 2 cases from the same donor in which the early diagnostic and therapeutic approach prevented potentially fatal IA.

## 3 Discussion

Our episodes exemplify the potential risk of IA transmission to recipients from donors with risk factors for invasive fungal infections. The donor we report had received broad-spectrum antibiotics and corticosteroids and developed IA affecting the lung, CNS, and kidneys. There are 2 major types of donor-derived infections: those that are easily detected by donor and recipient screening (cytomegalovirus, tuberculosis and toxoplasmosis) and those not easily detected despite routine donor screening [6].

Donor-related fungal infections are uncommon and reported as small case series or case reports [4], [5], [7], [8], [9]. *Candida* is the most common cause and is not always clearly related to the donor [7].

Transmission of filamentous fungi through organ donation, although infrequent, occurs under specific clinical circumstances [4], [5]. The main associated clinical circumstances in donors are: being a transplant recipient, brain hemorrhage as the cause of death, prolonged ICU stay and mechanical ventilation, near drowning, and transplant tourism [3], [7]. Our donor fulfilled 3 of them.

Most donor-derived filamentous fungal infections occurred in kidney transplant recipients (91%) [3]. Presentation of a donor-derived fungal infection can produce vascular complications at the graft site (65%), acute graft dysfunction (43%), and fever of unknown origin (39%) [10]. Vascular complications due to donor-derived fungal infections appeared suddenly with a severe or fulminant presentation. The most common findings were graft-related arterial pseudoaneurysm, arterial or venous thrombosis, graft-site abscess and fungal balls, and hemorrhagic shock due to arterial rupture [3]. In kidney transplant, acute renal failure was the most common form of graft dysfunction (90%) [3], [7].

In the cases we report, the growth of *A. fumigatus* (>100cfu/mL) was reported 24 h after donor death in the BAL sample. There was no previous calcofluor white stain. These samples are not routinely processed, unless at the clinician's request. As with most cases reported in literature, the present cases underwent graft explantation. It is important to mention that calcofluor white stain is mandatory in potential donors with a high risk of IA.

As these cases demonstrate, aggressive diagnostic efforts must be undertaken if there is suspicion of donor-derived fungal graft infection. Percutaneous graft biopsy for histopathology and fungal culture, graft imaging, and early surgical exploration are all part of the diagnostic approach. Our patients also had positive BDG titers in serum and urine, and this can purportedly be used in future episodes as early diagnostic tools.

Donor-related IA is associated with severe consequences, such as graft loss (83%) and a high mortality rate (17%) [3], which, in our opinion, justify the rapid medical and surgical intervention.

Invasive fungal infections represent a diagnostic and therapeutic challenge. However, awareness of the risk of donor-derived fungal infections may help clinicians to properly identify donors potentially capable of transmitting them and facilitate timely recognition of transmission to recipients.
